# Outer membrane vesicles in *Vibrio* species: Roles in biofilm formation and pathogenesis

**DOI:** 10.15698/mic2026.06.877

**Published:** 2026-06-02

**Authors:** Kumari Shambhavi, Durg Vijai Singh

**Affiliations:** Department of Biotechnology, School of Earth, Biological and Environmental Sciences, Central University of South Bihar, Gaya 824236, India

**Keywords:** *Vibrio* species, Outer Membrane Vesicle, Extracellular Vesicles, Membrane Vesicles, Bacterial Extracellular Vesicles, Biofilm Formation, Vaccine, Immunity

## Abstract

Outer membrane vesicles (OMVs) have been increasingly recognized as common mediators of bacterial physiology in Gram-negative bacteria, including *Vibrio* species. The degree and function of OMV production can differ among strains and even within a single species. The secretion of OMVs is a prevalent trait among many *Vibrio* species, particularly in pathogenic organisms such as *Vibrio cholerae*, *Vibrio vulnificus*, and *Vibrio parahaemolyticus*. The OMVs released by these organisms are often associated with infection, transport of virulence factors into host cells, defense against stress, biofilm formation, flagella rotation, transportation of active enzymes, signaling molecules in the surrounding environment, and facilitating bacterial translocation. All of these are advantageous to the bacteria. These OMVs also possess immunogenic properties that regulate the innate and adaptive immune responses, which are beneficial to host cells. Few species, such as *Vibrio ordalii*, *Vibrio coralliilyticus*, *Vibrio natriegens Vibrio alginolyticus, and Vibrio europaeus,* have been recently studied for the first time that secrete OMVs; future research is necessary to determine any other activities that these vesicles may possess beyond those that are now documented.

## INTRODUCTION

All living species secrete cellular components across their plasma membranes, which is a necessary function that enables them to interact with their surroundings. Secreting vesicles, which are spherical, nanoscale objects produced from the lipid membranes of the cell surface, are one method by which cells achieve this 1, 2. Gram-negative bacteria frequently release membrane-bound structures called extracellular vesicles (EVs) 3. EVs generally contain the same proteins, RNAs, DNAs, metabolites, and lipopolysaccharides (LPS) as their parent cell; however, some data suggest that specific proteins are enriched 4, 5. EVs exhibit a wide range of shapes 6 and membrane structures, with single or double membranes being the most common 7. Their diameter often ranged from 20 to 200 nm 8. Membrane vesicles (MVs) in Gram-negative bacteria are formed from endolysin-triggered explosive cell lysis, which is frequently brought on by genotoxic stress or from blebs of the outer membrane. The functions of MVs are scavenging nutrients, exporting bioactive molecules, neutralizing phages and antibiotics, and exerting bactericidal effects, as well as delivering virulence factors and toxins to host cells, and showed inflammatory and immunomodulatory effects, which are determined by their composition 9.

Outer membrane vesicles (OMVs) are increasingly recognized as common mediators of bacterial physiology in Gram-negative bacteria. Due to their unique molecular composition, OMVs are particularly potent effectors in inter-organismal interactions. These effects in bacterial pathogenesis have been thoroughly investigated 10. Human cholera is a diarrheal illness caused by the Gram-negative bacterium *Vibrio cholerae*. The cholera toxin (CT), a strong enterotoxin produced by this organism that colonizes the small intestine of humans, is the leading cause of diarrheal illness 11. Cholera vaccinations are necessary because *Vibrio cholerae* persists in water bodies, which will continue to hinder human efforts to eradicate the disease despite infrastructural improvements 12.

While diverse classes of MVs are produced across all domains of life, this review focuses on OMVs produced specifically by Gram-negative bacteria, with an emphasis on both native and engineered membrane vesicles shed by *Vibrio* species and their roles in biofilm formation and pathogenesis. We highlight the use of bacterial membrane vesicles (BEVs) of *Vibrio cholerae* in cholera vaccine research as an emerging therapeutics.

## TYPES OF MEMBRANE VESICLES

In Gram-negative bacteria, there are two pathways for the formation of MVs 13, 14: (i) blebbing of the outer membrane (B-type MVs), (ii) simultaneous curling and self-annealing of broken membrane fragments during explosive cell lysis (E-type MVs) 15. Genotoxic stress induces the expression of prophage-derived endolysins, leading to bacterial peptidoglycan layer disruption followed by explosive cell lysis 15–17. Outer membrane blebbing caused by cell envelope disruptions such as unbalanced peptidoglycan biosynthesis, the buildup of denatured proteins, or the intercalation of hydrophobic substances into the outer membrane produced OMVs. Outer-inner membrane vesicles (OIMVs) are formed 18 by blebbing mechanism through autolysin-mediated thinning of the bacterial peptidoglycan layer, followed by the protrusion of the inner membrane into the periplasm. After that, the vesicle and the surrounding outer membrane are pinched off from the cell surface, releasing the contents of the cytoplasm 19.

Prophage-mediated explosive cell lysis is induced in *Shewanella vesiculosa* M7T, enhancing the heterogeneity of both single- and double-layer membrane vesicles. The sequenced DNA fragments from the MVs encompassed the complete genome, validating this explosive cell lysis mechanism. The discovery of explosive cell lysis-derived double-layer MVs lead to the proposal to designate them as explosive OIMVs, which is different from the blebbing OIMVs 20. Vesicle formation begins with disruption of peptidoglycan layer causing explosive cell lysis which is induced by phase derived endolysin. These fragmented membrane then self-anneal leading to cells round up and bursting, generating either explosive outer-inner membrane vesicles (EOIMVs) or explosive outer membrane vesicles (EOMVs) 19.

## COMPOSITION OF MEMBRANE VESICLES

The MVs synthesis originates from the outer membrane in Gram-negative bacteria 21, 22. These disparities in membrane origin lead to discrepancies in their vesicles composition. For example, Gram-positive MVs lacks LPS, but it is exclusively present in Gram-negative bacteria 23. Historically, bacterial membrane vesicles (BMVs) has been studied keeping in mind their functions, particularly in relation to their pathogenicity. BMVs contained proteins and lipids, small genetic molecules, including messenger RNA (mRNA), small RNA, micro RNA (miRNA) and small interfering (siRNA), and genomic DNA, and contribute to pathogenesis by evading host immune cells, silencing specific gene expression and suppressing the immune response 24–27.

Most known OMVs from Gram-negative bacteria are loaded with LPS that are structurally identical to those of the parent bacteria 28–30. These OMVs are characterized by the presence of cell wall components, LPS, enzymes, various proteins, secondary metabolites, DNA molecules, and a diverse range of RNA molecules, including transfer RNA (tRNA), mRNAs, non-coding RNAs, and ribosomal RNA (rRNA) fragments 31–34. The OMVs that closely resembles with outer membrane contain phosphatidylethanolamine (PE), a major phospholipid identified in *Helicobacter pylori* followed by cardiolipin. Other phospholipids identified in *Helicobacter pylori* are OMVs comprised of lyso-PE, phosphatidylglycerol (PG), lyso-PG, phosphatidylcholine (PC), and lyso-PC 35. *Helicobacter pylori* OMV exhibits significant variation in protein composition, perhaps influenced by strain genotypes, varying bacterial growth circumstances, development phases, OMV synthesis techniques, and the sensitivity of mass spectrometry methods employed for proteomic investigation 36. A recent study indicated that the size of OMVs influences their protein cargo, with smaller OMVs exhibiting a markedly reduced quantity and diversity of proteins relative to bigger OMVs, which is predominantly encapsulated in *Helicobacter pylori* adhesins 37. Furthermore, genetic materials such as extracellular DNA (eDNA) and short non-coding RNA (sncRNA) have been identified in OMVs from several Gram-negative bacteria 28, 38. The proliferation of *Helicobacter pylori* in acidic environments reduced both the quantity and size of OMVs produced. Furthermore, OMVs generated under acidic growth conditions exhibited elevated levels of protein, DNA, and RNA cargo compared to those produced under neutral conditions. Proteomic research comparing the proteomes of OMVs with those of their parental bacteria revealed substantial disparities in the enrichment of beta-lactamases and outer membrane proteins, indicating that varying growth conditions influences OMV composition 39.

Gram-negative bacteria produce OIMVs, likely through mechanisms analogous to those of OMVs. OIMVs possess an outer and an inner membrane, enclosing periplasmic and cytoplasmic contents, including DNA 17. Phage-mediated lysis can generate E-OMVs in Gram-negative bacteria. Explosive cell lysis releases substantial quantities of DNA fragments, which simultaneously produced membrane fragments that may capture, therefore, constituting an additional mechanism for the generation of DNA-containing micro-vesicles 15. The fragmentation of chromosomal DNA within MVs suggests that the DNA present in these MVs originates from deceased cells 40, 41. E-type MVs exhibited elevated level of DNA content and showed higher incidence of horizontal gene transfer compared to OMVs 42–44. Fragmented OMVs can enclose extracellular plasmid DNA, suggesting this as an alternate route for DNA packaging into OMVs. This technique closely resembles MV production by explosive cell lysis, where recircularizing membrane fragments sequester the liberated DNA. The examination of total MV-associated DNA indicates that the sequences encompassing the complete genome 15, 44 are present in OMVs; however, studies examining the luminal DNA fractions have noted enrichment for specific chromosomal regions 24, 45.

## RELATIONSHIP BETWEEN OUTER MEMBRANE VESICLES AND BIOFILM

A polymicrobial community of microorganisms embedded in a matrix made of their own Extracellular Polymeric Substances (EPS) and attached to or associated with a biotic or abiotic surface known as a biofilm 46. Biofilm formation is a microbial survival strategy employed by bacteria to shield themselves from the host immune system and to enable fluctuations in their microenvironment, including pH, temperature, UV radiation, nutrient scarcity, and antimicrobial assaults, thereby facilitating successful infections despite adverse environmental conditions 47. The EPS matrix is the defining component of microbial biofilms, comprising 97% water and encompassing all principal classes of macromolecules, such as polysaccharides, proteins, and nucleic acids. It may also include peptidoglycan, lipids, phospholipids, and other cellular constituents 48–50.

MVs are other crucial elements of the EPS matrix in many bacteria, which perform distinct tasks within the biofilm based on their cargo. MVs can facilitate adhesion processes, cell-to-cell aggregation, defense mechanisms, nutrient acquisition, and chemical delivery, thereby contributing to the maintenance of biofilm homeostasis 48–50.

Quorum-sensing (QS) signal molecules can regulate biofilm formation; however, there is no known evidence that OMVs can encapsulate and mediate these signal molecules to influence biofilm regulation. Scientists isolated and purified OMVs containing *Pseudomonas* quinolone signal (PQS) generated by *Pseudomonas aeruginosa* and examined the impact of OMV-mediated PQS on biofilm formation and on their architecture 51. OMV-mediated PQS facilitated biofilm proliferation, resulting in the elongation, deformation, and interconnection of cells within the biofilm with adjacent cells.

## OMV PROMOTE BIOFILM FORMATION

Numerous investigations suggest that several MV components are crucial for biofilm formation and maintenance; however, their specific roles are challenging to clarify due to compositional complexity of MVs. A 22-kDa protein was implicated in biofilm development for a particular strain of *Helicobacter pylori* 52. MV from *Vibrio cholerae* El Tor C6706 contained the DegP protein, which facilitated biofilm formation 53, and the mere existence of these vesicles in *Francisella* biofilm indicates their role in its development 54.

Quorum signalling is recognized for its regulation of *Pseudomonas aeruginosa* biofilm development, and the PQS governs the biofilm formation 55–58. PQS functions not only as a signalling molecule but also regulates the production of OMVs 59–63. PQS stimulates OMV synthesis via a biophysical process facilitated by advantageous interactions with LPS in outer leaflet of the outer membrane 60, 64. Although numerous studies have shown association of PQS with the production of *Pseudomonas aeruginosa* biofilm, its role in all phases of biofilm development remains uncertain. PQS and OMVs were produced at peak levels during biofilm dispersion. PQS biosynthesis and receptor mutants have reduced dispersion relative to the wild type. The observed dispersion defect was rectified in a PQS receptor mutant by the introduction of exogenous PQS, reinforcing the hypothesis that the signalling-independent role of PQS (namely, OMV induction) significantly contributes to *Pseudomonas aeruginosa* biofilm dispersion. The data suggest that OMVs may facilitate biofilm dispersion by transporting enzymes that degrade key matrix components 65.

Some proteins in *Aeromonas* biofilms have amino acid sequences homologous to those of functional proteins in the outer membranes of Gram-negative bacteria, as confirmed by sodium dodecyl sulphate-polyacrylamide gel electrophoresis (SDS-PAGE). This observation suggests that the outer membrane components of *Aeromonas* strains may influence development of biofilm. Most *Aeromonas* strains discharged OMVs beyond the cells, as visualized in electron microscopy. Purification of OMVs from various *Aeromonas* strains and evaluating their biofilm-forming effects showed that, except for one strain that did not develop biofilms, OMVs increased biofilm formation in a dose-dependent manner. These data suggest a link between biofilm formation by *Aeromonas* strains and the production of OMVs by bacterial cells 66.

A cohort of cold-adapted Antarctic Gram-negative bacteria was used to determine if their biofilm formation capability correlates with their ability to create MVs and secrete extracellular adenosine triphosphate (ATP). There was no link between biofilm development and these two parameters in most of the examined strains. Only *Shewanella vesiculosa* M7T released elevated quantities of extracellular ATP, and its MVs significantly enhanced both the rate and amount of biofilm development. A significant fraction of the foreign ATP was encapsulated into MVs, thereby safeguarding it from apyrase treatment 44.

The ability of uropathogenic *Escherichia coli,* a Gram-negative pathogenic bacterium, to form persistent, antibiotic-resistant biofilms is required for their existence in the host urinary tract. These biofilms possessed proteins auto-transporter of bacterial surface proteins like Antigen 43 (Ag43). The auto-transporter Ag43 in *Escherichia coli* facilitates bacterial cell aggregation and develops biofilm by promoting self-association among adjacent bacteria that express Ag43. *Escherichia coli* expressing Ag43 generated MVs that displayed Ag43 on their surface and demonstrated increased adherence to *Escherichia coli.* The incorporation of Ag43-containing MVs into Ag43-expressing *Escherichia coli* markedly improved biofilm development 67.

A comprehensive proteomic approach to identify proteins associated with the biofilm matrix of the reference strain *Burkholderia multivorans* C1576, a clinical isolate with cystic fibrosis, revealed that the bacteria also release OMVs into the biofilm matrix. The proteome study revealed that OMVs are highly concentrated in outer membrane proteins and siderophores, and cytoplasmic and membrane-bound proteins are widely distributed throughout the matrix 68.

## OMVS INHIBIT BIOFILM FORMATION

In recent years, various groups of scientists have shown the decisive antibacterial action of OMVs via small molecules, surfactants, and enzymes 69–74. Studies demonstrated that oxidase-positive, Gram-negative *Burkholderia thailandensis* OMVs inhibit drug-sensitive and drug-resistant bacteria and fungi. A combination of tetra- and penta-acylated lipid A species in *Burkholderia thailandensis* reduces LPS-mediated endotoxicity 75. *Burkholderia thailandensis* OMVs contain closely associated antimicrobial substances like peptidoglycan hydrolases, hydroxy-methyl naphthoquinone (HMNQ), and long-chain rhamnolipid 76, and has a role to play in disrupting biofilm formation, a key aspect of bacterial pathogenesis and a potential therapeutic target 77.

*Pseudomonas aeruginosa* is an opportunistic bacterial pathogen and is capable of inducing both acute and chronic infections in humans. A clinical isolate of *Pseudomonas aeruginosa* was assessed for the expression of the *algD* and *ppyR* genes, in the presence of OMVs from *Escherichia coli* Nissle1917 (EcN OMVs) whose role was demonstrated in biofilm formation. EcN OMVs resulted in a more pronounced reduction of *algD* and *ppyR* expression relative to the control group. EcN OMVs encompass a diverse array of chemicals that can modulate the genes associated with biofilm development. The treatment with EcN OMVs dramatically diminished *Pseudomonas aeruginosa* biofilm formation, underscoring their beneficial effect in reducing biofilm development, which could potentially be harnessed for therapeutic purposes 78. *Pseudomonas aeruginosa* peptidase, PaAP, a leucine aminopeptidase, is significantly expressed during infection and within biofilms, and it associates with bacterial OMVs, which are known to play a role in virulence mechanisms across various Gram-negative species and constitute a significant component of the biofilm matrix. The deletion of PaAP in a clinical *Pseudomonas aeruginosa* context alters the composition of biofilm microcolonies, leading to increased cellular density and reduced matrix polysaccharide content. Moreover, OMVs from PaAP-expressing strains, not from PaAP alone but in conjunction with OMVs derived from the PaAP deletion strain, could restore this phenotype. OMVs from PaAP-expressing strains could induce protease-mediated biofilm dissociation, resulting in alterations in matrix and colony composition. Ultimately, OMVs may facilitate the detachment of biofilms established by both non-self-*Pseudomonas aeruginosa* strains and *Klebsiella pneumoniae*, another respiratory pathogen 79.

OMVs have the intriguing effect to enhance the resistance to polymyxin B in *Pseudomonas aeruginosa*. The overall level of gene transcription in OMVs can diminish the effect of polymyxin B in *Pseudomonas aeruginosa*. Both OMVs and sub lethal doses of polymyxin B inhibits and influences the level of transcription of QS system related genes in *Pseudomonas aeruginosa*. Moreover, the suppression of these genes lead to reduction in the virulence factors, bacterial motility, and biofilm formation 60. EVs in *Pseudomonas aeruginosa* biofilms secreted during exponential growth phase enhanced biofilm growth. In contrast, EVs when secreted in biofilms during death/survival phase (D-EVs) can effectively inhibit/eliminate *Pseudomonas aeruginosa* PAO1 biofilms up to 4.8 log10 CFU/cm ${}^{2}$ . The inhibitory efficacy of D-EVs against *Pseudomonas aeruginosa* biofilms grown for 96 hours was enhanced in the presence of 10–50 μ M Fe ${}^{3+}$ ions. Proteomic analysis suggests that inhibition involves an iron-dependent ferroptosis mechanism 80.

Three types of OMVs were isolated and described from biofilm-forming *Yersinia enterocolitica* Y1083, grown at 15 ${}^{\circ }$ C, 28 ${}^{\circ }$ C, and 37 ${}^{\circ }$ C, which have different yields and protein contents. Next, co-culturing OMVs with *Yersinia enterocolitica* showed that OMVs suppressed biofilm formation but not growth. OMVs also prevented the development of *Salmonella enteritidis* biofilms. LPS decreased bacterial motility and biofilm-related gene expression (*pgaABC*, *motB*, *flhBD*), and inhibited biofilm formation in several bacteria 81.

## OMV PRODUCTION BY *VIBRIO ORDALII*

*Vibrio ordalii*, a fish pathogen, produces and releases OMVs 82. SEM revealed the average size of OMV in *Vibrio* strains to be 0.215 ± 0.6 μ m; however, dynamic light scattering showed sizes ranging between 0.19 and 1.8 μ m. OMV patterns showed similarities in SDS-PAGE; however, they differ between total and external protein. The most noticeable bands while comparing *Vibrio ordalii* ATCC 33509 T and Vo-LM-18 were in the total protein, and the OMV profiles showed the highest degree of similarity between proteins of 37 kDa and 50 kDa sizes. Haemolytic enzyme activity was detected in pure OMVs, which may be important during *Vibrio ordalii* infection. To further our understanding of the proteome of Chilean *Vibrio ordalii* strain Vo-LM-18 and its OMVs, which are known to be virulent 83, possessed 2242 and 1755 proteins, respectively. The proteins that were identified in Vo-LM-18, belong to 644 distinct proteins, 156 unique proteins, and 1596 common proteins. The primary classifications for the OMVs were analogous to those present in bacteria, which were specifically belong to cytoplasmic and cytoplasmic membrane proteins. Functional annotation revealed the presence of 37 biochemical pathways in OMVs of strains Vo-LM-18 and 28, and proteins belonging to transport, transcription, and pathogenicity were prevalent in both of them. Their OMVs showed an elevated level of virulence-associated proteins, comprising those involved in iron and heme absorption, flagellum assembly, heme group-associated proteins, and protein production. In the inaugural study, the proteome analysis identified Repeats-in-Toxin (RTX) toxin in a Vo-LM-18 and its vesicle.

## OMV PRODUCTION BY *VIBRIO CHOLERAE*

The primary virulence factor of pathogenic *Vibrio cholerae* is the CT, an A-B type (AB5) bacterial toxin released via the type II secretion system. The predominant form of CT is secreted in association with OMVs and is found solely within these vesicles. Pure OMVs from the toxic *Vibrio cholerae* O395 disclosed the presence of spherical-shaped vesicles with a size range of 20–200 nm. Fluorescently labelled OMVs interacted with intestinal epithelial cells through the CT receptor, internalizing them and raising the concentration of cyclic adenosine monophosphate (cAMP). OMVs may therefore be a crucial means of delivering CT to epithelial cells 8. CT toxin cannot interact with monosialotetrahexosylganglioside (GM ${}_{1}$ ) receptors on the surface of host cells, and OMVs are transported to the host cells through a mechanism that does not rely on GM ${}_{1}$ . These observations suggest an additional, non-competitive pathway for the secretion and delivery of CT, in addition to the extensively studied Type II secretion system 84. Despite being physiologically active, OMV-encapsulated CT is not present in an AB5 form; instead, two enzymatic A-subunit polypeptides of CT (CTA) are enclosed within the OMVs 85.

Ninety proteins were identified in OMVs of *Vibrio cholerae* when grown in an environment that activated the Toxin-coregulated pilus (TCP), virulence regulatory protein (ToxT), which regulates the virulence regulon, as determined by trypsin digestion and mass spectrometry. Thirteen OMV-associated proteins may be potential targets for antibacterial drugs, as they are essential for cell development. DegP protease was one of the 12 non-essential OMV proteins required for intestinal colonization in rabbits. The significance of DegP in the integration of nine proteins into OMVs, including those involved in the development of biofilm matrix and different substrates of the type II secretion system, was demonstrated by comparative proteomics of a DegP mutant. These findings thus suggested that DegP plays a crucial role in determining the OMVs composition and characteristics such as intestinal colonization, the operation of a healthy type II secretion system, and the development of the biofilm matrix 53. DNA analysis of vesicular DNA from *Vibrio cholerae* O395, indicated there are ToxR binding sites that are more abundant in OMV than in whole-cell DNA. There is a positive association between ToxR enrichment and the proportion of adenine and thymine bases (AT-content). DNA-binding protein genes *hupA, hupB, ihfB, fis,* and *ssb*; membrane protein genes *OmpU, OmpK,* and *OmpV* are abundant in the vesicle fraction. Additionally, a negative correlation was observed between mRNA enrichment and transcript length, indicating that mRNA inclusion in vesicles may be a size-dependent process 86.

A novel short non-coding RNA (sRNA) gene, *vrrA*, has been identified in the *Vibrio cholerae* O1 strain A1552. A *vrrA* mutant exhibits excessive production of OmpA porin, and 140 nucleotide *VrrA* RNA inhibits *ompA* translation by base-pairing with the 5 ${}^{\prime }$ region of the mRNA. The RNA chaperone Hfq is not strictly essential for *VrrA* function; nonetheless, *vrrA* gene expression necessitates the membrane stress sigma factor, σ E, indicating that *VrrA* influences *ompA* in response to periplasmic protein folding stress. OmpA levels exhibited an inverse correlation with the quantity of OMVs, and *VrrA* enhanced OMV formation to a degree similar to the absence of OmpA. *VrrA* is the inaugural sRNA identified to regulate OMV development 87. An *ompA* mutant of *Vibrio cholerae* exhibited prolonged survival compared to wild-type *Vibrio cholerae* when cultured independently. Co-cultivation with *Acanthamoeba castellanii* enhanced the survival of both bacterial strains, whereas the OmpA protein had no effect on attachment, engulfment, or survival within the amoeba. Nonetheless, the co-cultivation of the *ompA* mutant of *Vibrio cholerae* diminished the survival of *Acanthamoeba castellanii*, and this bacterial strain produced a greater quantity of OMVs than the wild-type *Vibrio cholerae*. Treatment of amoeba cells with OMVs derived from the *ompA* mutant markedly reduced the viable counts of the amoeba cells. In conclusion, these studies underscore a regulatory role of *ompA* in the survival of *Vibrio cholerae* and OMVs, indicating it as a significant virulence factor for this bacterium in relation to eukaryotes in the environment 88.

**Figure 1:**
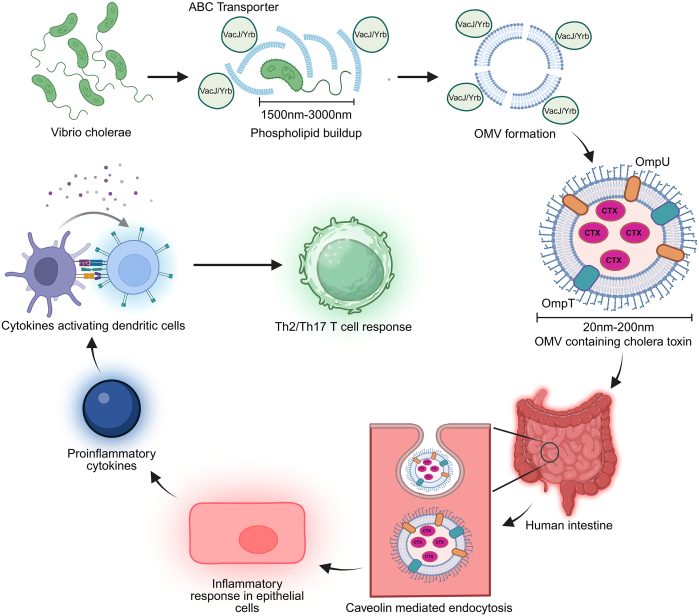
Delivery of CT to intestinal epithelial cells. The phospholipid transporter VacJ/Yrb controls vesicle formation from outer membrane phospholipid build-up. Bacteria exchange cell surface components through outer membrane vesiculation during mammalian infection to survive. Human intestinal epithelial cells use caveolin-mediated endocytosis to internalize these vesicles, which need surface effectors OmpU and OmpT. OMV-associated CT is shielded from intestinal proteases, extending the cAMP increase and toxin activity. Upon internalization, OMVs induce proinflammatory cytokines and activate dendritic cells that increase Th2/Th17 T cell responses in epithelial cells via NOD1-dependent pathways.

Phospholipid build-up in the outer membrane drives the pruction of vesicles, which is controlled by the phospholipid transporter VacJ/Yrb. It was demonstrated that VacJ/Yrb is repressed early during mammalian infection using the facultative human pathogen *Vibrio cholerae* 89. This phenomenon promotes vesiculation, which facilitates rapid bacterial surface exchange and adaptation to the host environment. The composition of the bacterial membrane changes rapidly in the hyper-virulent strain, which also shows improved intestinal colonization fitness. Faster glycine modified LPS accumulation and reduction of outer membrane porin OmpT, which gives resistance to bile and host-derived anti-microbial peptides, respectively, are two examples of this adaptation. Therefore, during mammalian infection, bacteria increases their chance of survival by exchanging components of their cell surface through outer membrane vesiculation. Human intestinal cell lines are quick to absorb vesicles produced by the therapeutically relevant El Tor biotype of *Vibrio cholerae*. Outer membrane porins of *Vibrio cholerae*, specifically OmpU and OmpT, have been reported essential surface effectors on OMV for cellular uptake, with caveolin-mediated endocytosis being the identified uptake mechanism 90 (Figure 1).

Additionally, OMVs from *Vibrio cholerae* cultured under virulence-promoting conditions serve as effective carriers for delivering bioactive CT to intestinal epithelial cells. Unlike free CT released through the type II secretion system, the CT associated with OMVs is shielded from degradation by intestinal proteases. Collectively, these findings suggest that OMV-associated CT can persist in the intestinal tract for extended periods, maintaining its toxic effects, as indicated by a sustained increase in cAMP levels within the intestinal tissue 104. Upon internalization, OMVs stimulate inflammatory responses in epithelial cells through Nucleotide-binding oligomerization domain-containing protein 1 (NOD1)-dependent pathways, leading to the induction of proinflammatory cytokines and the subsequent activation of dendritic cells that facilitate Th2/Th17 T cell responses 91 (Figure 1).

**Figure 2:**
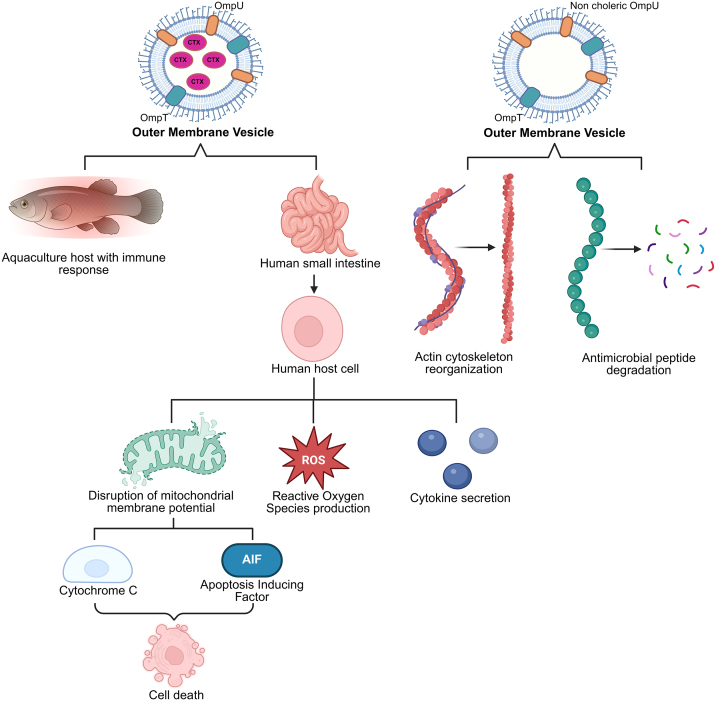
OmpUs’ interaction with the host cell. OmpU interacts with host cells to increase pathogenicity, triggering signalling cascades that promote the release of cytokines and the generation of ROS. Translocating to mitochondria, it alters mitochondrial membrane potential, releasing cytochrome C and AIF, which causes caspase-independent programmed cell death in immune and epithelial cells. OmpU causes an immunological reaction in several aquaculture hosts. Non-cholera OmpU causes actin cytoskeleton rearrangement in the host cells and confers resistance to antimicrobial peptides.

An experimental evolution experiment have identified the number of genes that restore motility in the presence of a sub-inhibitory concentration of polymyxin B. Altogether, mutation in five genes were identified in three variants originating from two distinct parental strains, A1552 and MO10: (i) *ihfA*, encoding for a subunit of the integration host factor (IHF); (ii) *vacJ (mlaA)* and (iii) *mlaF*, both associated with the maintenance of lipid asymmetry (Mla) pathway; (iv) *dacB,* encoding for a penicillin-binding protein 4 (PBP4) involved in cell wall synthesis; and (v) *ccmH*, encoding for a c-type cytochrome maturation protein. MO10-derived variants with mutations in *vacJ, mlaF,* and *dacB* secrete larger quantities of membrane vesicles that titrate polymyxin B, enhancing bacterial survival and potentially mitigating its effects on the bacterial envelope while contributing to flagellum retention and motility 92. The engagement of OMVs with intestinal epithelial cells resulted in the upregulation of proinflammatory cytokines, including IL-8 and granulocyte-macrophage colony-stimulating factor (GM-CSF), as well as chemokines such as CCL2, CCL20, and thymic stromal lymphopoietin (Figure 2). This process occurred through the activation of the mitogen-activated protein kinase (MAPK) and Nuclear factor kappa-light-chain-enhancer of activated B cells (NF- κ B) signalling pathways, a process dependent on NOD1 91.

Similar to other bacterial species, *Vibrio cholerae* incessantly emits BEVs from its surface, which have recently been characterized for their function during in vivo colonization. Nonetheless, between epidemic outbreaks, *Vibrio cholerae* endures in a biofilm state for prolonged durations in aquatic reservoirs, hence augmenting environmental adaption and host transmission. Ebenberger SP et al. examined the impact of *Vibrio cholerae* BEVs on biofilm development, an essential characteristic for ex vivo survival 93. Unlike BEVs derived from planktonic cultures, their findings indicated that physiological amounts of BEVs from dynamic biofilm cultures promote *Vibrio cholerae* biofilm development, which may be associated with a proteinaceous component. Comparative proteome investigations of planktonic and biofilm-derived BEVs revealed a previously uncharacterized outer membrane protein as a prominent component of dynamic biofilm-derived BEVs, which was determined to facilitate the BEV-dependent augmentation of biofilm development. As a result, this protein was designated as outer membrane-associated biofilm facilitating protein A (ObfA). Extensive molecular investigations revealed ObfA as an inhibitory regulator of HapR activity. HapR is a crucial transcriptional regulator of the *Vibrio cholerae* quorum-sensing cascade, serving as a potent repressor of biofilm development and pathogenicity. ObfA mutants have consistently shown diminished biofilm formation and lower colonization fitness, and influence HapR not through the conventional quorum-sensing system, but rather through the Csr-cascade, which modifies the expression of the short regulatory RNAs CsrC and CsrD 93.

Recent research showed that the *Vibrio cholerae* biofilm matrix also contained OMVs, which were linked to OMPs and other biofilm matrix proteins, suggesting that they were involved in biofilm matrix formation. OmpU was the most common outer membrane protein in the matrix; its removal altered the biofilm architecture by increasing the production of *Vibrio* polysaccharides (VPS). Single-cell force spectroscopy revealed that the matrix components RbmA, RbmC, Bap1, VPS, and OmpU are critical for biofilm formation and contribute to cell surface adhesion, applying to varying degrees of effectiveness, with VPS demonstrating the highest efficiency and Bap1 the lowest 94. OmpU is a significant outer membrane protein of *Vibrio* species that functions as a crucial virulence factor via various interactions with host cells. OmpU acts as an adhesin, facilitating bacterial adherence to host cells via specific N-terminal motifs, notably amino acid sequences 90–101 and 173-192 95. The protein promotes bacterial invasion through its interaction with β -integrins and alters the host actin cytoskeleton 96, 97. In addition to adhesion, OmpU has considerable cytotoxic effects on host cells. It initiates caspase-independent programmed cell death by translocating to the mitochondria, altering mitochondrial membrane potential, and facilitating the release of cytochrome C and apoptosis-inducing factor (AIF) 98 (Figure 2). OmpU additionally facilitates cytokine secretion, enhances ROS generation, and confers resistance to antimicrobial peptides 97. The complex relationships render OmpU crucial for effective *Vibrio* colonization and pathogenicity across a diverse range of hosts, including humans and aquatic organisms (Figure 2).

The wild-type *Vibrio cholerae* strain C6706 secretes PrtV via OMV mediated type II secretion system. The biological activity of OMV-associated PrtV was altered morphology and cell detachment of human ileocecal carcinoma (HCT8) cells when treated with OMVs from the wild-type *Vibrio cholerae* strain C6706. In contrast, there was no morphological changes seen after treating cells with OMVs extracted from *prtV* isogenic mutant 99.

*Vibrio cholerae* secretes the calcium-dependent trypsin-like serine protease (VesC) and zinc-dependent hemagglutinin protease (HAP) using the type 2 secretion system. These proteases are released in association with OMVs and are in an active form in the epithelial cells of the human gut. Whereas OMV-associated VesC showed a haemorrhagic fluid response in the mouse ileal loop (MIL) assay, caused necrosis in Int407 cells, and showed increased level of interleukin-8 (IL-8) in T84 cells, but showed reduced activities with OMVs when isolated from VesC mutant strain. Similarly, OMV-associated HAP induced dose-dependent apoptosis in Int407 cells in addition to enterotoxic activity in the MIL assay. These findings thus suggest that serine protease VesC is involved in the intestinal colonization of *Vibrio cholerae* strains in adult mice 100. *Vibrio cholerae* expressed two active rhomboid proteases, namely universally conserved intramembrane rhomboid protease (GlpG) and rhomboid protease rhombosortase (RssP). These proteases cleave a common substrate at distinct locations, resulting in the processed protein that is localized differently. GlpG cleavage produces and secrete completely soluble VesB, then RssP cleaves and directs VesB towards the bacterial cell surface and then to OMVs 101. OMV-associated HapA impairs the integrity of tight and adherence junctions in intestinal epithelial cell more efficiently than its pure form, indicating that interaction of HapA with OMVs significantly enhanced its pathogenic effects. The researchers demonstrated the absorption of *Vibrio cholerae* OMVs by epithelial cells and their specific degradation of essential junctional proteins, such as claudin, ZO-1, and β -catenin 102.

The defence mechanism known as secreted OMVs protects *Vibrio cholerae* from phage predation. This OMV-mediated suppression is dependent on the phage receptor 103. The virulence factor *Vibrio cholerae* cytolysin (VCC) is translocated by OMVs released by the bacteria. Target eukaryotic cells are lysed by VCC, a pore-forming toxin that creates transmembrane oligomeric β -barrel channels. OMV-associated VCC elicited the target cell’s autophagy response, and autophagy may function as a cellular defence mechanism against an OMV-associated bacterial virulence factor 104. To facilitate colonization, *Vibrio cholerae* releases OMVs, and activates miR-146a, which is achieved by weakening the epithelial innate immune response and averting mucosal inflammation triggered by stimuli such as VCC 105.

Immunization using OMVs generated from *Vibrio cholerae*, which has been genetically detoxified, triggers the production of O-antigen antibodies that bind to the sheathed flagellum and effectively prevent movement. A species-specific immune response was elicited by immunization against *Vibrio cholerae* or enterotoxigenic *Escherichia coli* (ETEC) OMVs. Still, a high-titre, protective immune response against both pathogens was stimulated by a combination of two OMV species. Further immunizing against ETEC colonization with *Vibrio cholerae* OMVs produced an as-yet unidentified, cholera toxin B-subunit (CTB)- independent defence mechanism 106.

OMVs secreted by *Vibrio cholerae* are a suitable option for creating a vaccine. OMVs coated with Eudragit, an enteric polymer, and encased in chitosan-tripolyphosphate (TPP) nanoparticles made using an ion gelation technique. OMVs that were encapsulated or free were used to immunize OMVs loaded onto nanoparticles (NP-OMVs). When the novel vaccination was given through the mucosa, it was able to prevent infection completely 107. A new vaccination option for Severe Acute Respiratory Syndrome Coronavirus 2 (SARS-CoV-2) based on bacterial OMVs was designed 108. Genetic modifications have been made to *Vibrio cholerae* and ETEC to enhance their production of detoxified OMVs adorned with the receptor-binding domain (RBD) of the SARS-CoV-2 Spike protein. The most effective neutralizing activity and the highest titres against the SARS-CoV-2 spike protein were observed with an alternative immunization regimen using RBD-decorated OMVs from *Vibrio cholerae* and ETEC, respectively. These findings demonstrate that there is a variety of vaccines that use OMVs expressing heterologous antigens in the donor bacteria.

## VIBRIO CHOLERAE OMVS AS A POTENTIAL CHOLERA VACCINE

For the first time, when mice inoculated with purified OMVs produced from *Vibrio cholerae* via the intranasal, intragastric, or intraperitoneal routes induced high-titer, specific, and persistent immune responses against a range of antigens contained in the OMVs 109. Analysis of newborns’ sera and stomach content showed high concentrations of immunoglobulin G (IgG) against the OMV. While prenatal transfer alone inhibited colonization, the transfer of immunoglobulin to neonates via milk was sufficient to completely protect the neonates from colonization by *Vibrio cholerae* 110. LPS is the primary antigen that protects against OMVs. Although serotype-specific antigens predominate, mucosal immunization with OMVs from Inaba or Ogawa strains offers considerable cross-serotype protection against *Vibrio cholerae* colonization. Immunizing against a combination of O1 and O139 OMVs does not offer cross-serogroup protection. As antibodies derived from OMV-immunized mice decrease Vibrio cholerae motility in vitro, reflecting its tendencies in vivo and provide neonatal protection that may involve motility inhibition rather than considerable bacterial mortality 111. Neonates challenged with hyper-infectious *Vibrio cholerae* had much less colonization after being vaccinated with OMV 112. *Vibrio cholerae* induces the development of protective immunity in the rabbit model 113. Compared to both the live and heat-killed bacteria, OMVs were found to be substantially less reactogenic. A cholera vaccine candidate’s LPS-directed immune response was identified using LPS mutations 114. LPS-modified OMVs produced by *Vibrio cholerae* were shown to be the primary protective antigen of the vaccine candidate. Through genetic alteration of lipid A, endotoxicity can be reduced without compromising the immunogenic potential of the vaccine candidate. The O-antigen was identified as the primary immunogenic structure and offers a defense mechanism that inhibits motility, thereby preventing colonization from succeeding.

The production of toxin-coregulated pilus associated protein F (TcpF), a secreted colonization factor, by *Vibrio cholerae* O1 and O139 has sparked interest in it as a potential protective antigen in the development of subunit cholera vaccines. In comparison to TcpF alone, a TcpF + CTB mixture, or CTB alone, 115 was assessed for immunogenicity and protective effectiveness of a TcpF holotoxin-like chimera (TcpF-A2-CTB) after intraperitoneal immunization. Anti-TcpF IgG was substantially evoked by vaccination with the TcpF-A2-CTB chimera than by immunization with the other antigens.

Bishop et al. observed neutrophil recruitment at the height of infection, along with the elevation of NOS-2, KC, macrophage inflammatory protein (MIP-2), IL-6, and IL-17a 126. In a backdrop of wild-type cells, the accessory toxins RtxA and Alpha-hemolysin A (HlyA) did not appear to have any effect on neutrophil recruitment. Significant reductions in the innate response to *Vibrio cholerae* deleted for cholera toxin encoding phage (CTX ϕ ) and a part of RtxA were seen, indicating a potential for either RtxA or CTX ϕ -carried genes in the absence of CT CTX ϕ 116.

Immunoglobulin, especially IgG, in the milk of mucosally immunized dams provides immunity against OMVs. *Vibrio cholerae* organisms are rendered immobile by anti-OMV IgG, allowing them to transit through the small intestine without colonization. IgG selectively binds to the O-antigen of *Vibrio cholerae*, impeding motility through both in vitro and in vivo tests. IgG’s bivalency is necessary for motility inhibition but not for binding to the O-antigen 117. Sinha et al. isolated OMV from several serotypes and combined it with five highly pathogenic *Vibrio cholerae* strains 118. Oral immunization with cholera pentavalent OMVs (CPMVs) produced specific B and T cell responses against *Vibrio cholerae* after four doses. Mice inoculated with CPMVs produced serum IgG, IgA, and IgM, as well as mucosal sIgA, which lasted for a long time. Additionally, the mice’s spleen produced a higher percentage of CD4+ T cells. Long-term immunity may be induced by oral immunization with *Vibrio cholerae* OMVs, particularly when combined with killed whole-cell (KWC) 119. Active metabolites of vitamin A, All-trans Retinoic acid (ATRA), have adjuvant and anti-inflammatory effects on mucous membranes. In vitro and in vivo Toll-like receptor 2 (TLR2)-mediated pro-inflammatory responses generated by CPMVs were considerably inhibited by pre-treatment with ATRA. After receiving two doses of oral immunization with CPMVs (75 μ g), pre-treated ATRA significantly enhanced the mucosal immune response and protective efficacy 120.

Three major virulence factors in *Vibrio cholerae* with heightened immunogenicity are ctxB (toxin binding to eukaryotic cells), tcpA (bacterial colonization), and ompW (coding for a highly conserved extracellular protein). Zareitaher T, et al. evaluated the stability and effectiveness of a chimeric gene encoding *ompW*, *tcpA*, and *ctxB* (OTC) as a vaccine against *Vibrio cholerae* using DNA and protein-based vaccine candidates. High survival rates against fatal infection were offered by the OTC design, which also markedly decreased bacterial burdens 121.

Liu R, et al. found that OmpV augments the intestinal colonization and cytotoxicity of *Vibrio cholerae* 122. The expression of OmpV is induced by the TCS CarSR, with phosphorylated CarR directly attaching to the *ompV* promoter in response to HD-5 123. OmpV, present in the outer membrane, promotes bacterial adherence to the intestinal epithelium, while OmpV in BEVs aids in the internalization of these vesicles by host cells. This procedure enhances the uptake of CT into host cells, thereby stimulating proinflammatory cytokine synthesis. Based on this information, Liu R et al. designed a drug targeting OmpV as a prospective treatment for cholera 122. By leveraging the structural and functional attributes of OmpV, they employed computational-aided drug design (CADD) 124 to develop the antibacterial drug C607-0736, which efficiently inhibits OmpV-mediated bacterial adhesion and CT delivery. Additionally, it was observed that C607-0736 can markedly impede the colonization of *Vibrio cholerae* O1/O139 and OmpV-expressing *Vibrio parahaemolyticus* strains carrying *ompV* in animal studies. These findings indicate that C607-0736 may be effective in treating infection caused by pandemic *Vibrio cholerae* 122.

## OMV PRODUCTION BY *VIBRIO PARAHAEMOLYTICUS*

*A* significant halophilic bacterium, *Vibrio parahaemolyticus* causes a wide range of diseases in aquatic animals and can also cause catastrophic illness in humans who consume seafood such as fish, shrimp, and shellfish. Using an immunological assay and mass spectrometry analysis, novel immunogenic OMPs were identified in *Vibrio parahaemolyticus*. Four of the nine OMPs, namely LptD, VP0802, VP1243, and VP0966, showed relatively low abundance in OMP profiles and were found to exhibit immunogenicity for the first time. Bioinformatics investigation suggests that VP0802 belongs to the OprD protein family and adopts a β -barrel shape, is highly conserved among major *Vibrio* species, and is found to be highly immunogenic, which provides robust protection against *Vibrio parahaemolyticus* infection 125. In *Vibrio parahaemolyticus,* the OmpW, OmpA, long-chain fatty acid transporter protein, outer membrane receptor protein, putative uncharacterized protein VP0167, maltoporin (lamB), polar flagellin B/D, agglutination protein, peptidoglycan-associated lipoprotein, and membrane-bound lytic transglycosylase (MltA)-interacting protein MipA can be classified as ultraviolet C-induced (UVC)-stress proteins 126. Isolation of OMV from *Vibrio parahaemolyticus* acute hepatopancreatic necrosis disease (VpAHPND) and its analysis yielded a total of 645 proteins, encompassing virulence factors, immunogenic proteins, outer membrane proteins, bacterial secretory proteins, ribosomal proteins, proteases, and iron regulatory proteins. Moreover, Gene Ontology (GO) and Kyoto Encyclopaedia of Genes and Genomes (KEGG) annotations revealed that the proteins identified in VpAHPND-OMV are involved in diverse biological processes, genetic information processing, immunity, and other functions. Additionally, proteome analysis of VpAHPND-OMV revealed the presence of the toxin proteins PirAvp and PirBvp, which are associated with VpAHPND pathogenicity 127.

*Vibrio parahaemolyticus* OMVs (Vp-OMVs), which induce immune response in aquatic species, were used to investigate their impact on the innate immunity of mud crabs (*Scylla paramamosain*) 128. The Vp-OMVs confirm the presence of vesicles exhibiting the characteristic OMV shape and dimensions, which were ingested by host cells via clathrin-mediated endocytosis, leading to cytotoxicity, elevated mortality, tissue vacuolation, and diminished cell viability. Additionally, Vp-OMVs, which include LPS, activate the TLR2 signalling pathway, thus enhancing the synthesis of antimicrobial peptides. Further, internalized Vp-OMVs induced inflammasome activation and mitochondrial malfunction, leading to inflammation and death. These findings highlight the crucial role of Vp-OMVs in the pathogenesis of *Vibrio parahaemolyticus* infections in mud crabs. Acute hepatopancreatic necrosis disease, a major bacterial pathogen affecting the aquaculture of *Litopenaeus vannamei* (Pacific white shrimp), was caused by *Vibrio parahaemolyticus*. VpAHPND-OMVs showed effect on the innate immune responses of *Litopenaeus vannamei*. Notable changes were in immune-related enzyme activities, such as lysozyme, superoxide dismutase, alkaline phosphatase, and glutathione S-transferase. These findings indicate that VpAHPND-OMVs can swift within a brief timeframe and substantially augment the activity of immune enzymes. This study suggests that VpAHPND-OMVs may exert a dual regulatory impact on shrimp, initially stimulating the immune system but potentially leading to immunosuppression with prolonged exposure 129.

A 20-strand transmembrane β -barrel domain formed by the *Vibrio parahaemolyticus* porin gene (Vp-porin) in *Vibrio parahaemolyticus* is conserved mainly in *Vibrio* species. Vp-porin depletion inhibits flagellar synthesis, leading to motility impairment, while also contributing to virulence and various biological functions, including the maintenance of membrane integrity, promotion of motility, and conferring resistance to antibiotics 130.

## OMV PRODUCTION BY *VIBRIO FISCHERI*

*Vibrio fischeri* can only produce robust biofilms when the symbiosis polysaccharide (*syp*) locus is activated, which is done by overexpressing the senior kinase RscS+. Both RscS+ and control cells’ culture supernatants could be used to purify OMVs; the former released two to three times as many OMVs as the latter. This increase was dependent on the existence of an intact *syp* locus. An RscS+ strain that has deleted the *sypK* gene, which codes for a putative oligosaccharide translocase, showed decreased OMV production. Deletion of *degP*, which affects OMV synthesis in other bacteria, resulted in reduced OMV production and a decrease in the cell’s ability to form biofilms. The presence of the *syp* locus is necessary for the overexpression of RscS+ to drive the creation of OMVs, and the OMVs generated under these conditions contain molecules that are antigenically different and may be a modified form of LPS 131.

When *Vibrio fischeri* moves from its ambient reservoir to the acidic environment of host tissues, it upregulates transcription of its primary outer membrane protein, OmpU. The OmpR, H-NS, and ToxR proteins control the differential expression of OmpU. A combination of dietary factors and transcriptional regulation controls OmpU levels in OMVs. In an OmpU-dependent way, *Vibrio fischeri* OMVs become more powerful stimulators of symbiotic host development when expressed at an acidic pH 10. OMVs are an essential component in numerous host-cell/microbe interactions. Prior investigations into the symbiotic relationship between *Euprymna scolopes* and *Vibrio fischeri* have demonstrated that within 12 hours of colonizing the crypts of the squid’s light organ, the symbionts initiate an irreversible program of host tissue development. OMVs produced by *Vibrio fischeri* significantly contribute to this process. The initial evident host response to the OMV is an augmented migration of macrophage-like cells, known as haemocytes, into surface epithelial tissues. It was demonstrated that exposing the squid to other *Vibrio* species does not elicit this trafficking; rather, the introduction of a high concentration of their OMVs, which can permeate the crypts, does. Through the manipulation of the time and introduction of OMV signal delivery, the haemocyte trafficking is fully activated only when *Vibrio fischeri*, the exclusive species capable of reaching and proliferating in the crypts, successfully establishes sustained colonization 132. Moriano-Gutierrez S, et al. reported that light-organ symbiosis between *Vibrio fischeri* and squid *Euprymna scolopes* is dependent on the bacterial sRNA Small stable RNA A (SsrA) 133. The OMV that the symbionts load with SsrA is specifically delivered into the epithelial cells surrounding the symbiont population in the light organ. Thus, the capacity of sRNAs produced by bacterial symbionts regulates not only their own activities but essential host responses as well that support homeostasis 133.

In vitro exposure to modelled microgravity (reduced gravity) has been demonstrated to increase the number of LPS released by the bacterial symbiont. The elevated rates of shedding observed under conditions of modelled microgravity are associated with an increased production of OMVs. Defective mutants of *Vibrio fischeri*, which are unable to produce and rotate their flagella, demonstrated a notable reduction in LPS shedding across all experimental conditions 134. However, despite the loss of motility, the levels of LPS are elevated under conditions of modelled microgravity. Additionally, modelled microgravity seems to impact the outer membrane integrity of *Vibrio fischeri*, because cells incubated under such conditions demonstrate heightened susceptibility to agents that disrupt the cell membrane.

## OMV PRODUCTION BY *VIBRIO SPLENDIDUS*

A common Gram-negative marine bacterium, *Vibrio splendidu*s, is responsible for illness in a variety of marine farmed animals 135. Song H et al. demonstrated for the first time that *Vibrio splendidus* secretes OMVs, carrying functional proteins that enhanced bacterial survival under various stresses 136. After OMV purification, the protein composition was analysed by proteomic analysis, which revealed the presence of approximately 120 proteins comprising outer membrane proteins, flagellins, ABC transporters, proteases, and iron-regulating proteins. These proteins were involved in bacterial motility, biofilm formation, and other cell membrane components 136.

The *aerAVs* gene encoding for an aerolysin of *Vibrio splendidus* was cloned and conditionally expressed in *Escherichia coli* to understand the function of aerolysin in the OMVs as virulence factors. Results of western blot analysis concluded that aerolysin is a virulence factor of *Vibrio splendidus*, which exhibits both haemolytic and cytotoxic activity, 137.

## OMV PRODUCTION BY *VIBRIO CORALLIILYTICUS*

*Vibrio coralliilyticus* DSM19607, a coral-associated strain, produced OMVs in culture. The purified OMVs from *Vibrio coralliilyticus* DSM 19607 showed inhibition of the bacteriophage SBM1 infection in the host, which can be impaired by elevated temperature. Additionally, the detection of these membrane vesicles in coral mucus suggests that it has potential for interactions between vesicles and phages in the natural environment. These findings thus suggest that OMVs play a regulatory role in the coral microbiome 138.

## OMV PRODUCTION BY *VIBRIO VULNIFICUS*

BEVs were isolated from *Vibrio vulnificus* cells growing under optimal conditions by density gradient ultracentrifugation. The diameter of the purified BEVs ranged from approximately 25 nm to 161 nm, as determined by dynamic light scattering measurements. Park et al. postulated that nucleotide sequences unique to RNAs packaged in BEVs might differ in a few ways from those of cellular RNA molecules 139. Using next-generation sequencing (NGS), the nucleotide sequence and sRNA abundances between the cellular fraction and BEVs were examined. The lengths of sRNA fragments from BEVs were noticeably shorter than those in the cytoplasm, even though there was no discernible difference in the nucleotide sequences of the sRNAs between the two groups.

When mice are infected with *Vibrio vulnificus*, significant levels of *Vibrio vulnificus* cytolysin-hemolysin (VvhA) are produced in vivo. This cytotoxin, in conjunction with RtxA1, enhances cytotoxicity. OMVs produced by this organism can kill host cells. In contrast, OMVs isolated from VvhA-null mutants do not produce cytotoxicity suggesting the role of VvhA in the mediation of this mechanism. Furthermore, OMV-mediated VvhA transport is inhibited by cholesterol sequestration in host cells, suggesting that VvhA-containing OMVs interact with cholesterol on the host cell surface. Moreover, studies on intracellular expression demonstrated that the N-terminal leucocidin domain of VvhA is responsible for its cytotoxicity 140.

OMVs generated by *Vibrio vulnificus* were shown to include outer membrane lipoproteins but not inner membrane lipoproteins. The aspartate at position ＋2 of mature lipoproteins can serve as an inner membrane retention signal 141. While investigating the effects of *Vibrio vulnificus* outer membrane vesicles (Vv-OMVs) on host cells, 142 they found stimulation of INT407 epithelial cells with purified Vv-OMVs. They also observed alterations in morphology and release in lactate dehydrogenase (LDH) levels. Cells after treatment with OMVs showed detachment of cells without LDH release, thus exhibits different characteristics compared to cytotoxic cell detachment observed in *Vibrio vulnificus* infection. This study thus suggests that the proteolytic function of a serine protease present in Vv-OMVs may contribute to the pathogenicity of *Vibrio vulnificus* by facilitating bacterial translocation. The expression of capsular polysaccharide affects the Vv-OMV production 143.

## OMV PRODUCTION BY *VIBRIO NATRIEGENS*

The first evidence that *Vibrio natriegens* can produce OMVs throughout the exponential growth phase was reported 144. The first proteomic snapshot of OMVs and related whole samples across different growth phases revealed that the OmpA24 protein represents a promising candidate for delivering heterologous protein fusion cargo into OMV.

## OMV PRODUCTION BY *VIBRIO HARVEYI*

The proteins found in the exosomes secreted by *Vibrio harveyi* possessed virulence factors such as lipase and phospholipase, and exhibited vital bacterial functions such as carbon metabolism, fatty acid biosynthesis, and antibiotic production 145. The hydrophobic QS molecule Cholerae Autoinducer-1 (CAI-1), a long-chain amino ketone, is packaged into the OMV of the marine pathogen *Vibrio harveyi*. The ability to package CAI-1 into OMV may help in stabilizing the molecule in an aqueous environment and transport active enzymes and signalling molecules 146.

## OMV PRODUCTION BY *VIBRIO ANGUILLARUM*

Production of OMVs in *VIbrio anguillarum* O1, a significant fish pathogen responsible for vibriosis. They were generated during standard growth and manifested as spherical vesicle fractions. The protein composition of the OMVs resembled to that of the outer membrane proteins, containing 38-kDa predominant protein band of OMV designated as OmpU. The OMVs exhibited enzymatic activity of metalloprotease, hemolysin, and phospholipase, and induced the synthesis of proinflammatory cytokines, including TNF- α , IL-1 β , and IL-6 upon injection into the flounder *Paralichthys olivaceus* 147*.*

Lumpfish are also vulnerable to *Vibrio anguillarum* infection, and once infected, their immune response is triggered to prevent the progression of illness. EVs are crucial for initial immunological cellular communication. At 5- and 10-days post-infection (dpi), EVs were recovered from the serum of naïve lumpfish and lumpfish infected with *Vibrio anguillarum*. Electron microscopy, nanoparticle tracking, western blotting, and proteomic analysis of EVs revealed a spherical morphology with diameters ranging from approximately 30 nm to 300 nm. The number variance increased to a total of 395 proteins at five dpi. The proteins associated with the complement pathway/innate immunity, heme/iron binding, defense response to bacteria, apoptotic signalling pathway, and actin binding were upregulated. At the same time, proteins associated with ribonucleoprotein/ribosomal protein functions, transport and translation elongation factor activities, acute-phase responses, protein phosphorylation, and apoptotic processes were downregulated. *Vibrio anguillarum* infection converts the cargo of lumpfish EVs from metabolic proteins to immune-related proteins 148. Besides containing hemolysin and metalloprotease, the virulence factor in *Vibrio anguillarum* participates in essential bacterial functions, including carbon metabolism, fatty acid biosynthesis, and antibiotic synthesis 145.

## OMV PRODUCTION BY *VIBRIO SHILONII*

The coral pathogen *Vibrio shilonii* can transmit BEVs to convey messages to the holobiont organism 17. OMVs are produced in vitro by the well-researched coral pathogen *Vibrio shilonii* AK1. Transmission electron microscopy revealed that the pure vesicle fraction from *Vibrio shilonii* cultures displayed a vesicle chain-like morphotype, in addition to two types of vesicles with single or double membranes. Furthermore, alkaline phosphatase, lipase, and chitinase activity are present in the OMVs isolated from AK1 cultures incubated at both 20 ${}^{\circ }$ C and 30 ${}^{\circ }$ C, along with N-acyl homoserine lactone QS signals. *Vibrio shilonii* OMVs transport active enzymes, signalling molecules, and other proteins to their surroundings 149.

## OMV PRODUCTION BY *VIBRIO TASMANIENSIS*

*Vibrio tasmaniensis* LGP32, a facultative intracellular pathogen of oyster haemocytes, was demonstrated to release OMVs in both the external environment and within haemocytes. The OMV proteome of LGP32 is abundant in hydrolases (25%), including putative virulence factors like proteases, lipases, phospholipases, haemolysins, and nucleases. A significant caseinase/gelatinase, designated Vsp (vesicular serine protease), was identified as being exclusively secreted by OMVs, where it is encapsulated. Vsp was shown to contribute to LGP32’s virulence phenotype in experimental oyster infections 150.

## OMV PRODUCTION BY *VIBRIO ALGINOLYTICUS*

The production of biofilm acts as a significant virulence factor of *Vibrio alginolyticus* 151. Historically, bacterial biofilm research has primarily focused on transcriptional regulatory mechanisms that control virulence gene expression. For example, *rpoS* has been recognized as a crucial regulator of biofilm formation in *Vibrio alginolyticus.* The RNA interference (RNAi) method was employed to downregulate *rpoS* in *Vibrio alginolyticus*. Compared to wild-type *Vibrio alginolyticus*, RNAi-treated bacteria exhibited markedly reduced capacities for adhesion, proliferation, hemolysis, biofilm formation, motility, and pathogenicity. Simultaneously, variations in temperature, salinity, pH, and starvation significantly influenced *rpoS* expression 152. Recent studies have progressively emphasized the importance of sRNAs in the biofilm development process. Zuo Y, et al. showed that the virulence-associated Srna and Vvrr1 regulates the expression of the pyruvate kinase I-encoding gene, *pykF*, therefore affect biofilm formation in *Vibrio alginolyticus* 153. Another sRNA, Vvrr2, implicated in adhesion control, contributes to environmental adaptation through negative regulation. Its overexpression has been shown to disrupt the three-dimensional architecture of biofilms by altering the chemical composition of EPS 154.

The initial data indicated that *Vibrio alginolyticus* can produce OMVs 155. The study was sought to identify and purify OMVs from *Vibrio alginolyticus* and describe their properties. The validation of OMVs revealed that it had a protective roles for their cargo, which is similar to those noted in other bacterial OMVs 156. RNA sequencing revealed the presence of 10 most abundant sRNAs in OMVs’ nucleic acid cargo. These sRNAs were numbered in descending order of their relative abundance from Va_OMV_R1 to Va_OMV_R10. Of the target genes and regulatory pathways of sRNAs, the highly expressed (Va_OMV_R1) and signalling pathways (Va_OMV_R8) sRNA was selected for further study. The knockout and complementation of strains for these two sRNAs, were compared for their growth, motility, and biofilm formation. It was found that the absence of these sRNAs alters target gene expression, impacting the strain’s physiological capacities, particularly biofilm formation. The comparison of MVs isolated from wild-type and sRNA knockout strains indicated that wild-type strains can increase biofilm formation in dose-dependent manner in *Vibrio alginolyticus*, because these strains showed accelerated rate of biofilm formation leading to significant increase in biomass. Still, the absence of Va_OMV_R1 and Va_OMV_R8 reduces the biofilm formation.

## OMV PRODUCTION BY *VIBRIO EUROPAEUS*

*Vibrio europaeus* has emerged as a significant pathogen of bivalves in the past decade 157. They utilize type II, type III, and three distinct type VI secretion systems (T6SS1, T6SS2, and T6SS3) to expel virulence components into the extracellular milieu 158. Furthermore, OMVs, classified as a type 0 secretion system, represent a significant pathway for the secretion of these compounds into the extracellular milieu 159. Researchers did the extensive characterisation of the extracellular products and secretome of the mollusc pathogen *Vibrio europaeus*. A total of 108 distinct proteins were reported in the wild-type secretome, with the extracellular proteins, namely VemA, VepA, and GbpA, comprising about 70% of the total secreted protein. A substantial fraction of cytoplasmic proteins was also identified in the wild-type secretome, aligning with their secretion via OMVs observed using electron microscopy. Functional assays demonstrated that VemA is critical for virulence against Manila clam juveniles (*Ruditapes philippinarum*), while VepA and GbpA seem to have complementary or modulatory functions. Unexpectedly, the concurrent deletion of vemA, vepA, and gbpA genes prompted significant reprogramming of secretory system, resulting in release of 823 secretory proteins, which is about eightfold higher than the wild type, and displaying unique enzymatic activity. Notwithstanding its augmented array of virulence-associated proteins, the triple mutant exhibited the lowest pathogenicity in challenge analysis, indicating that the absence of the principal extracellular products initiates compensatory stress responses that alter secretion without reinstating the virulence phenotype. These findings indicate that VemA, VepA, and GbpA are crucial factors influencing virulence and secretory homeostasis in *Vibro europaeus*, highlighting an adaptive process that connects OMV-mediated secretion, proteome plasticity, and pathogenic potential in marine *Vibrio* species 160.

The key features of MVs that have been reported from various *Vibrio* species have been summarised (Table 1).

**Table 1:** Key features of MVs isolated from different *Vibrio* species .

Species	OMV characteristics	Major OMV cargo	Biological functions of OMVs	Relevance (pathogenesis, immunity, ecology)
*Vibrio ordalii*	Heterogeneous vesicles ( ∼ 0.19–1.8 μ m), strain-dependent profiles	Haemolytic enzymes, Virulence and transport proteins, RTX toxin 83	Haemolysis, iron and heme absorption	First identification of RTX toxin in *Vibrio ordalii* OMVs; Fish pathogenicity

*Vibrio cholerae*	20–200 nm; vesiculation enhanced during infection, stress, and membrane remodeling	CT (CTA subunits) 85; OmpU 86, 90/OmpT 90/OmpV 86; proteases (VesC/HAP 100, PrtV 99); sRNAs 87	Toxin delivery, immune modulation, biofilm development, antibiotic resistance, phage defense	OMVs are the predominant vehicle for CT delivery; central role in vaccine development

*Vibrio parahaemolyticus*	Typical spherical OMVs; production influenced by stress and pathogenic state	PirAvp/PirBvp toxins 127; OMPs 125; LPS, immunogenic proteins 129	Immune activation and modulation, inflammation	Aquaculture and human disease

*Vibrio fischeri*	OMV production enhanced by RscS–syp locus activation; pH-dependent	Modified LPS 131; OmpU 10; sRNAs (SsrA) 133	Symbiosis establishment, host tissue development	Squid–bacteria mutualism

*Vibrio splendidus*	OMVs produced in vitro; stress responsive	Aerolysin 137; flagellins, transporters, iron-regulatory proteins 136	Stress tolerance, virulence	Marine animal disease

*Vibrio coralliilyticus*	OMVs detected in culture and coral mucus	Phage-interacting components 138	Phage inhibition, microbial community regulation	Coral microbiome stability under thermal stress

*Vibrio vulnificus*	25–161 nm; actively released during growth, cholesterol-dependent uptake	VvhA cytolysin, RtxA1 140; serine proteases 143; sRNAs 139	Host cell killing, bacterial translocation	Severe human infections

*Vibrio natriegens*	OMVs produced throughout exponential phase	OmpA24 144	Protein transport	Biotechnological applications

*Vibrio harveyi*	Continuous OMV secretion in marine environments	CAI-1 146; lipase, phospholipase 145	Quorum sensing, enzyme transport	Marine pathogenicity and communication

*Vibrio anguillarum*	30–300 nm; spherical OMVs during growth	OmpU, hemolysin, metalloprotease, phospholipase 147	Fish immune activation, Cytokine induction	Fish pathogen; Host EV response

*Vibrio shilonii*	Multiple OMV morphotypes (single/double membrane)	Enzymes (lipase, chitinase), QS molecules 149	Host-holobiont signaling	Coral bleaching pathogen

*Vibrio tasmaniensis*	OMVs released extracellularly and intracellularly	Hydrolases, vesicular serine protease (Vsp) 150	Intracellular virulence	Oyster pathogen

*Vibrio alginolyticus*	OMVs produced during growth; enriched in regulatory RNAs	sRNAs (Va_OMV_R1–R10) 156	Biofilm regulation, environmental adaptation	Aquaculture pathogen

*Vibrio europaeus*	OMV-associated secretome expansion	VemA, VepA, GbpA 160	Virulence regulation, secretion homeostasis	Emerging bivalve pathogen

## BIOLOGICAL FACTORS INFLUENCING *VIBRIO* OMV RESEARCH

The comparison of OMV size, composition, and function among several *Vibrio* species is confounded by significant variation in experimental methodologies, bacterial strains, and physiological settings. The examined papers demonstrate how these factors influences reported outcomes and highlight the necessity for careful interpretation.

## VARIATIONS IN STRAIN-SPECIFIC CHARACTERISTICS AND PATHOGENICITY PROFILES

Numerous *Vibrio* species analyzed demonstrated significant strain-dependent variations in OMV composition. Proteomic investigation of *Vibrio ordalii* demonstrated significant disparities between the reference strain ATCC33509 ${}^{\text{T}}$ and the Chilean virulent isolate Vo-LM-18, notably the unique detection of RTX toxin in the latter strain and its OMVs 83. This indicates that OMV cargo represents the pathogenicity repertoire and genetic composition of specific strains rather than characteristics at the species level. In *Vibrio cholerae*, commonly utilized strains including O395, A1552, C6706, and MO10 exhibit variations in toxin synthesis, regulatory mechanisms, and membrane remodelling pathways 86, 92, 99, which account for the discrepancies in OMV-associated toxins, proteases, and nucleic acid content observed in various investigations.

## IMPACT OF GROWTH CONDITIONS AND HOST-RELATED SIGNALS

OMV production is acutely responsive to environmental variables, a phenomenon consistently noted in the examined research. Vesiculation in *Vibrio cholerae* is augmented under situations that promote virulence, such as bile exposure, polymyxin B stress, or intestinal infection, which coincides with the suppression of the VacJ/Mla lipid asymmetry pathway. These conditions produce OMVs that are enriched in CT, outer membrane porins, and immune-modulatory factors 90–92, while OMVs obtained from conventional laboratory growth may be devoid of these characteristics. Similarly, biofilm-derived OMVs in *Vibrio cholerae* and *Vibrio fischeri* significantly vary from planktonic vesicles, because biofilm-associated OMVs contain proteins such ObfA that actively facilitate biofilm development and enhance colonization fitness 10, 93.

## VARIABILITY IN VESICLE DIMENSIONS AND CHARACTERISATION TECHNIQUES

Reported OMV sizes exhibit significant variability among *Vibrio* species, indicative of both biological diversity and methodological discrepancies. For example, *Vibrio ordalii* OMVs varied from approximately 0.2 μ m to 1.8 μ m, contingent upon the application of SEM or dynamic light scattering 83, but *Vibrio cholerae* and *Vibrio vulnificus* OMVs were generally documented in the nanometer scale 8, 139. Discrepancies may result from variations in vesicle aggregation, purification rigor, and analytical resolution, highlighting the necessity for consistent characterization methods.

## STRATEGIES FOR OMV PURIFICATION AND CARGO ASSIGNMENT

Variations in OMV isolation procedures likely account for disparate proteomic and functional outcomes. Research utilizing density gradient ultracentrifugation (e.g., *Vibrio vulnificus*, *Vibrio cholerae*) demonstrates precise enrichment of virulence factors like VvhA cytolysin or CT 8, 139, whereas less rigorous techniques may co-isolate flagellar components, soluble poisons, or cellular detritus. This is especially pertinent for protease-rich species like *Vibrio tasmaniensis* and *Vibrio anguillarum*, where the enzymatic activity associated with OMVs may partially represent contaminated extracellular proteins 147, 150.

## DEPENDENCE ON FUNCTIONAL ASSAYS AND HOST MODELS

The biological impacts of *Vibrio* OMVs are frequently evaluated utilizing various host models, such as mammalian epithelial cells, fish, shrimp, mollusks, corals, and symbiotic squid. For instance, *Vibrio parahaemolyticus* OMVs elicit robust immunological activation in mud crabs and shrimp 128, 129, while *Vibrio fischeri* OMVs facilitate host tissue growth in squid 132. These divergent outcomes reflect genuine biological specialization but also underscore that OMV “virulence” or “benefit” is context-dependent and cannot be generalized across hosts. Furthermore, differences in OMV dose normalization and exposure duration may explain reports of immune stimulation versus immunosuppression, particularly in aquaculture models.

## CONSEQUENCES FOR INTER-STUDY COMPARISON

The examined literature indicates that the composition and function of OMVs in *Vibrio* species are influenced by strain identification, environmental circumstances, purification methods, and host context. Apparent discrepancies among studies—such as variations in toxin encapsulation, immunogenicity, or vesicle dimensions—are thus more likely to indicate experimental variability rather than biological contradiction. Recognising these limitations is essential for accurately interpreting OMV functions and for designing reproducible studies that can guide vaccine development, antimicrobial strategies, and ecological models of *Vibrio* biology

## CONCLUSION AND PERSPECTIVES

OMVs are increasingly recognized as common mediators of bacterial physiology in Gram-negative bacteria, including *Vibrio* species. Specifically, Gomez-Gil B, et al. 161 reported 131 species of *Vibrio*, while Sawabe T, et al. 162 noted 142 species in the Vibrionaceae family. Ceccarelli D, et al. confirmed 130 confirmed *Vibrio* species, with about a dozen known to cause human infections 163. The OMV production and function can differ among *Vibrio* species and even among various strains within a single species. The OMV secretion is a prevalent trait in many *Vibrio* species, particularly in pathogenic species such as *Vibrio cholerae*, *Vibrio vulnificus*, and *Vibrio parahaemolyticus*. The OMVs released by the *Vibrio species* are often associated with infection, transport of virulence factors in host cells, defense from stress, biofilm formation, flagellar rotation, transportation of active enzymes, signaling molecules in the surrounding, and facilitating bacterial translocation, which are advantageous to the bacteria, in addition to carrying immunogenic proteins that regulate the innate and adaptive immune response. Recently, additional *Vibrio* species, including *Vibrio splendidus*, *Vibrio coralliilyticus*, *Vibrio natriegens*, and *Vibrio europaeus*, have been examined for their OMV secretion. However, further research is required to ascertain any additional functions these vesicles may have beyond the currently documented activities. Despite substantial progress in characterizing OMV production and function across *Vibrio* species, several critical gaps remain that limit mechanistic understanding and translational application. Addressing these gaps will be essential for advancing OMV-based therapeutics, vaccines, and ecological models of *Vibrio* biology. Current studies employ heterogeneous OMV isolation protocols, ranging from simple differential centrifugation to density gradient purification, often without rigorous assessment of vesicle purity. This makes it difficult to distinguish true OMV cargo from co-purifying extracellular proteins, flagellar fragments, or cell debris. Researchers should develop and adopt standardized workflows that include density gradient purification, protease protection assays, and quantitative normalization (e.g., lipid or particle counts). Standardization would improve reproducibility, enable meaningful cross-species comparisons, and provide a reliable foundation for OMV-based vaccine and drug development. Most mechanistic studies rely on a limited number of laboratory strains (e.g., *Vibrio cholerae* O395 or A1552), despite clear evidence that OMV cargo and function vary dramatically among strains, as shown for *Vibrio ordalii*, *Vibrio cholerae*, and *Vibrio parahaemolyticus*. Comparative studies using panels of clinical, environmental, and hypervirulent strains, coupled with genomic and lipidomic profiling, are needed. This would clarify which OMV traits are conserved versus strain-specific, improving the ecological relevance of OMV studies and guiding rational vaccine antigen selection. While numerous toxins, proteases, and regulatory RNAs have been identified in *Vibrio* OMVs, the molecular mechanisms governing selective cargo packaging remain poorly defined. Research should prioritize identifying genetic determinants of cargo sorting, including roles of lipid asymmetry pathways (VacJ/Mla), envelope stress responses ( σ E), and rhomboid proteases. Understanding cargo selection would enable targeted engineering of OMVs for therapeutic delivery and help explain how *Vibrio* adapts OMV function to specific environmental or host conditions. Many OMV studies rely heavily on in vitro cell culture models, which may not fully recapitulate host complexity. In vivo data are uneven across species, with strong evidence in *Vibrio cholerae* but limited validation in other pathogenic and symbiotic *Vibrio* species. Greater use of physiologically relevant infection models (e.g., fish, shrimp, coral, squid, and mammalian systems) combined with OMV-deficient mutants. In vivo validation is essential for distinguishing biologically relevant OMV functions from experimental artefacts and for translating findings into applied interventions. OMVs have been reported to both stimulate and suppress host immune responses, particularly in aquaculture species exposed to *Vibrio parahaemolyticus* OMVs. The determinants of these opposing outcomes are unclear. Systematic studies varying OMV dose, exposure duration, host developmental stage, and immune status are required. Resolving this ambiguity will improve disease management strategies in aquaculture and help predict OMV-driven immune modulation during infection. OMVs have been implicated in biofilm formation, quorum sensing, and phage defense, and their broader ecological functions in marine microbial communities remain largely unexplored. Field-based and microcosm studies are required to examine OMV-mediated interactions among bacteria, phages, and eukaryotic hosts under natural conditions, which would integrate OMVs into models of marine nutrient cycling, microbial competition, and pathogen emergence. With improved standardization and mechanistic resolution, *Vibrio* OMVs are poised to become a unifying framework linking bacterial physiology, pathogenesis, and environmental function.

## CONFLICT OF INTEREST

The Authors declare that they have read the contents of the paper and do not have any competing interests.

## ABBREVIATIONS

Ag43 – Antigen 43

AIF – apoptosis-inducing factor

ATP – adenosine triphosphate

ATRA – all-trans retinoic acid

BEVs – bacterial extracellular vesicles

BMVs – bacterial membrane vesicles

CAI-1 – cholerae autoinducer-1

cAMP – cyclic adenosine monophosphate

CPMVs – cholera pentavalent membrane vesicle

CT – cholera toxin

CTB – cholera toxin B-subunit

CTXϕ – cholera toxin encoding phage

D-EVs – death/survival phase

dpi – days post-infection

EcN OMVs – *Escherichia coli* Nissle 1917 OMVs

EPS – extracellular polymeric substances

ETEC – enterotoxigenic *Escherichia coli*

E-type MVs – explosive-type MVs

EVs – extracellular vesicles

IgG – Immunoglobulin G

IHF – integration host factor

IL – Interleukin

LDH – lactate dehydrogenase

MIL – mouse ileal loop

Mla – maintenance of lipid asymmetry

mRNA – messenger RNA

MVs – membrane vesicles

NF-κB – nuclear factor kappa-light-chain-enhancer of activated B cells

NGS – next-generation sequencing

NOD1 – nucleotide-binding oligomerization domain-containing protein 1

ObfA – outer membrane-associated biofilm facilitating protein A

OIMVs – outer-inner membrane vesicles

OmpV – outer membrane protein V

OMVs – outer MVs

OTC – *ompW*, *tcpA*, and *ctxB*

PaAP – *Pseudomonas aeruginosa* aminopeptidase

PC – phosphatidylcholine

PE – phosphatidylethanolamine

PG – phosphatidylglycerol

PQS – *Pseudomonas* quinolone signal

RBD – receptor-binding domain

RNAi – RNA interference

ROS – reactive oxygen species

RssP – rhomboid protease rhombosortase

RTX – Repeats-in-Toxin

SARS-CoV-2 – severe acute respiratory syndrome coronavirus 2

SDS-PAGE – Sodium dodecyl sulphate-polyacrylamide gel electrophoresis

SEM – scanning electron microscopy

sRNA – short non-coding RNA

SsrA – small stable RNA A

T6SS – Type VI secretion systems

TCP – toxin-coregulated pilus

TcpF – toxin-coregulated pilus associated protein F

TcpF-A2-CTB – TcpF holotoxin-like chimera

TLR2 – Toll-like receptor 2

VCC – *Vibrio cholerae* cytolysin

Vo-LM-18 – *Vibrio ordalii* strain

VpAHPND – *Vibrio parahaemolyticus* acute hepatopancreatic necrosis disease

Vp-OMVs – *Vibrio parahaemolyticus* OMVs

Vp-porin – *Vibrio parahaemolyticus* porin

VPS – *Vibrio* polysaccharides

Vsp – vesicular serine protease

VvhA – *Vibrio vulnificus* cytolysin-hemolysin

Vv-OMVs – *Vibrio vulnificus* OMVs
